# Disparate roost sites drive intraspecific physiological variation in a Malagasy bat

**DOI:** 10.1007/s00442-021-05088-2

**Published:** 2021-12-24

**Authors:** Stephanie Reher, Hajatiana Rabarison, B. Karina Montero, James M. Turner, Kathrin H. Dausmann

**Affiliations:** 1 Functional Ecology, Institute of Zoology, Universität Hamburg, Hamburg, Germany; 2 Mention Zoologie et Biodiversité Animale, Faculté des Sciences, Université d’Antananarivo, Antananarivo, Madagascar; 3 Biodiversity Research Institute, Campus of Mieres, Universidad de Oviedo, Mieres, Spain; 4 Animal Ecology and Conservation, Institute of Zoology, Universität Hamburg, Hamburg, Germany; 5 Institute of Biomedical and Environmental Health Research, School of Health and Life Sciences, University of the West of Scotland, South Lanarkshire, Scotland, UK

**Keywords:** Torpor, Tropics, Physiological flexibility, Adaptive/facultative hyperthermia, Season

## Abstract

Many species are widely distributed and individual populations can experience vastly different environmental conditions over seasonal and geographic scales. With such a broad ecological reality, datasets with limited spatial and temporal resolution may not accurately represent a species and could lead to poorly informed management decisions. Because physiological flexibility can help species tolerate environmental variation, we studied the physiological responses of two separate populations of *Macronycteris commersoni*, a bat widespread across Madagascar, in contrasting seasons. The populations roost under the following dissimilar conditions: either a hot, well-buffered cave or within open foliage, unprotected from the local weather. We found that flexible torpor patterns, used in response to prevailing ambient temperature and relative humidity, were central to keeping energy budgets balanced in both populations. While bats’ metabolic rate during torpor and rest did not differ between roosts, adjusting torpor frequency, duration and timing helped bats maintain body condition. Interestingly, the exposed forest roost induced extensive use of torpor, which exceeded the torpor frequency of overwintering bats that stayed in the cave for months and consequently minimised daytime resting energy expenditure in the forest. Our current understanding of intraspecific physiological variation is limited and physiological traits are often considered to be fixed. The results of our study therefore highlight the need for examining species at broad environmental scales to avoid underestimating a species’ full capacity for withstanding environmental variation, especially in the face of ongoing, disruptive human interference in natural habitats.

**Supplementary Information:**

The online version contains supplementary material available at 10.1007/s00442-021-05088-2.

## Introduction

Discrete populations of widely distributed species may inhabit areas with different climatic conditions and, therefore, face contrasting environmental pressures. Prevailing conditions may also vary within and among seasons, making the complete ecological sphere of a species considerably broad. Among regions, individuals may seek out similar microhabitats and consequently display relatively low potential for withstanding environmental variation, while others can adapt locally or possess highly flexible physiological traits (Kobbe et al. 2011; Noakes and McKechnie 2020; Hume et al. 2020; van Jaarsveld et al. 2021). Differences on a population level, therefore, reflect a species' capacity for phenotypic variation and/or local adaptation, and may even hint at incipient speciation (Violle et al. 2012; Richardson et al. 2014). Since physiological capacity ultimately determines species' tolerance limits to abiotic factors, and their resilience to changes in their environment (Canale and Henry 2010; Bozinovic et al. 2011), climatic variation can be tolerated via individuals’ physiological flexibility. Brushtail possums *Trichosurus vulpecula* from arid habitat, for example, have a lower energy and water turnover and can dissipate heat more efficiently than conspecifics from mesic areas (Cooper et al. 2018). Similarly, big brown bat *Eptesicus fuscus* populations from higher latitudes have lower energetic costs at cooler ambient temperatures (*T*_a_) during torpor than their conspecifics closer to the equator (Dunbar and Brigham 2010). As a consequence, conclusions drawn from physiological data collected during only one season and/or from a single location may under- or overestimate a species’ full natural capabilities. This is especially critical in the face of ongoing human disruptive interference, when accurate research and reporting can help define suitable habitats that might serve as refugia, identify areas of increased risk and ensure the long-term viability of both populations and species (Irwin et al. 2010; Cooke et al. 2013; Rezende and Bacigalupe 2015; Cooper et al. 2018).

Bats are an ideal group for studying intraspecific physiological variation over broad environmental scales because many species are widely distributed, in some cases populating entire continents and thus a variety of habitats and environmental conditions. They are highly efficient at minimising energy expenditure and species from arid habitats can finely balance body water (e.g., Geiser and Stawski 2011; Klüg-Baerwald and Brigham 2017; Gearhart et al. 2020). Diurnal roost selection can limit exposure to unfavourable environmental conditions, such as weather extremes, help avoid predators (Fenton et al. 1994; Solick and Barclay 2006) and facilitate social interactions and reproduction (Kunz 1982; Willis and Brigham 2004). Moreover, both bat abundance and species distributions are correlated with roost availability (Humphrey 1975). Roosting sites are, therefore, a critical resource for many bats and can be an important determinant of extinction risk (Sagot and Chaverri 2015). Roosts range from well-buffered caves or crevices and tree holes, to other animals’ nests, anthropogenic structures, constructed leaf tents and completely exposed roosts in foliage (Kunz and Lumsden 2003). Some species or individuals rest strictly in only one type of roost, while others change depending on seasonal and/or life history requirements (e.g., overwintering, reproduction or rearing offspring; Kunz and Lumsden 2003). Understanding the seasonal preferences of discrete bat populations for a specific roost type, and the significance of variation in these different roosts’ microclimates, are therefore important steps for determining the full scope of a species’ ecology and physiology.

The endemic insectivorous Commerson’s leaf-nosed bat *Macronycteris commersoni* uses contrasting types of diurnal roost throughout its wide distribution in Madagascar (Goodman 2011). In a dry spiny forest in south-western Madagascar, *M. commersoni* roosts in a large colony in a hot cave with a highly stable microclimate (32 ± 0.1 °C at bat height, 98 ± 0.5% relative humidity; Reher et al. 2018) throughout the year that is buffered from external weather (Reher et al. 2019). In a western dry deciduous forest, on the other hand, the bats roost solitarily in the open vegetation among branches (Reher and Dausmann 2021). These tree roosts provide no insulation, leaving bats vulnerable to predators and the effects of external environmental extremes. Both habitats are located in the western formations in the driest zones of the island and are highly seasonal. During the harsh dry season precipitation and food availability are reduced for up to 9 months, and night-time *T*_a_ may occasionally drop to as low as 3–6 °C (Kobbe et al. 2011; Kappeler and Fichtel 2012). During the milder wet season food resources are more abundant and night-time *T*_a_ is milder (> 20 °C), but daytime *T*_a_ extremes regularly exceed 40 °C (Reher and Dausmann 2021). Such high *T*_a_ is challenging for the bats’ thermoregulatory systems, because when *T*_a_ > body temperature ( *T*_b_) only evaporative cooling can prevent hyperthermia, which is associated with high water expenditure (Mitchell et al. 2018) and may increase the risk of dehydration in a dry region.

To conserve water and energy, both populations use torpor (Reher et al. 2018; Reher and Dausmann 2021), which is a controlled state of metabolic depression (Geiser 2004; Heldmaier et al. 2004). In general, torpor is highly beneficial. However, it is unknown how torpor patterns expressed by *M. commersoni* vary with roost type and season, and are thus modified to cope with prevailing conditions. There is evidence that torpid metabolic rate (TMR) might not vary among bat populations resting at different roosts, even though evaporative water loss could (Klüg-Baerwald and Brigham 2017; Gearhart et al. 2020; McGuire et al. 2021). However, torpor duration, frequency and general patterns, as well as resting metabolic rate (RMR), may differ at different resting sites and among seasons (Stawski and Geiser 2011; Kobbe et al. 2014; Bethge et al. 2017; Boyles et al. 2020) to support the maintenance of homeostasis.

In our study, we investigated the energetic costs and benefits of physiological strategies used by *M. commersoni* to cope with different roosting conditions, i.e. roost type and season, and quantified intraspecific physiological variation therein. We compared torpor occurrence and timing in two habitats with differing roost types: a cave with near-constant environmental conditions and an open forest with fluctuating environmental conditions. We hypothesised that bats use torpor more often, and torpor bouts are longer, in the protected cave roost, especially during the resource-poor dry season. Given that variations in TMR in thermoconforming animals are mainly a function of *T*_a_ (Geiser 2004), we predicted a higher TMR in the forest bats when torpor is used in response to heat stress (Reher and Dausmann 2021), but no differences in TMR between habitats at the same *T*_a_. We also evaluated the energetic costs associated with the different roosting conditions by examining body condition and daytime resting energy expenditure (DREE).

## Methods

### Study sites

**Fig. 1:**
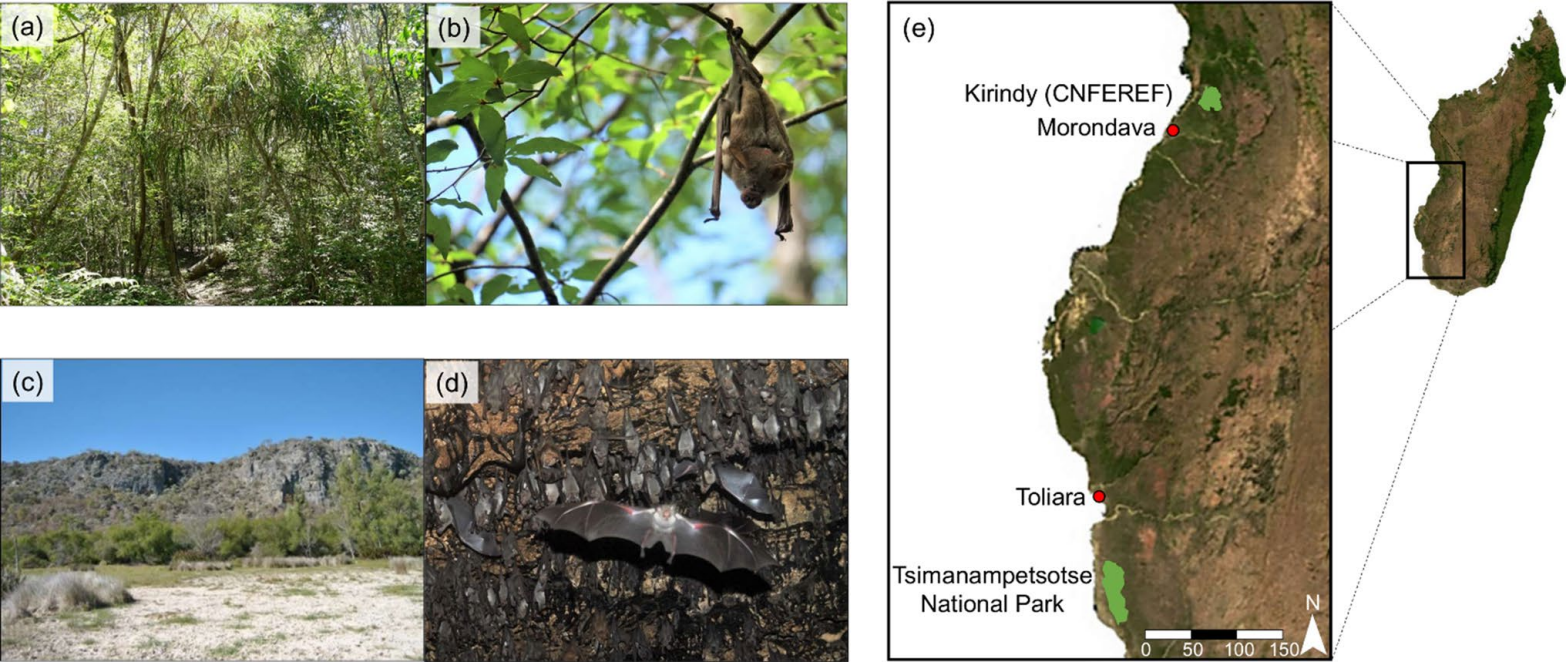
Typical habitat structure and diurnal roosts of *M. commersoni* at the two sites studied in Madagascar (**e**): a foliage roost (**b**) in dry deciduous dry forest (**a**) without caves in Kirindy Forest (CNFEREF); a cave roost (**d**) in dry spiny forest (**c**) in National Park Tsimanampetsotse

To study the effects of roosting conditions on bat metabolism, we collected data at two sites in Madagascar separated by about 380 km that differ in roost availability: a cave habitat in south-western spiny forest of Tsimanampetsotse National Park and a forest habitat in western dry forest in the Kirindy Forest/Centre National de Formation, d’Etudes et de Recherche en Environnement et Forestier (CNFEREF) (Fig. 1).

Tsimanampetsotse National Park encompasses a variety of different ecosystems including dry plains around a salt lake, dry spiny forest and a calcareous plateau with underground cave and stream systems (Reher et al. 2018, 2019; Fig. 1c, e). Although it experiences a cool dry season (April to October; daily *T*_a_ fluctuations between 16.2 and 32.5 °C) and a hot wet season (November to March; 24.4 °C–38.5 °C), this region is generally very dry year-round with only sporadic, unpredictable annual rainfall of less than 500 mm (or entire years without rain; Ratovonamana et al. 2013). Within the cave system, Andranolovy Cave (24.04585° S, 43.75396° E) is used by *M. commersoni* and several other bat species as a diurnal roost in the wet season and for overwintering during the dry season (Reher et al. 2019; Fig. 1d). It is the hottest and most humid cave in the region and cave conditions are highly stable year-round (Table 1) (Reher et al. 2018, 2019). The bats roost at a height of ~ 6 m, where cave temperature is 32.4 ± 0.1 °C and relative humidity is 98.3 ± 0.5%.

**Table 1:** Environmental conditions (ambient temperature and relative humidity) during the wet and dry seasons at each of the sites used by *M. commersoni*, measured at a height of 1.5 m above the ground, in Tsimanampetsotse National Park and Kirindy Forest (CNFEREF)

Region	Season	Variable	Mean	Range	Daily variation
Tsimanampetsotse National Park	Dry	Cave temperature (°C)	29.4 ± 0.1	29.3–29.6	0.1 ± 0.1
		Cave relative humidity (%)	98.8 ± 0.1	96.4–100.5	1.1 ± 0.3
	Wet	Cave temperature (°C)	30.6 ± 0.1	30.3–30.7	0.2 ± 0.1
		Cave relative humidity (%)	95.2 ± 0.1	93.8–96.6	1.6 ± 0.3
Kirindy Forest (CNFEREF)	Wet	Forest temperature (°C)	27.5 ± 1.2	21.2–41.7	13.8 ± 2.3
		Forest relative humidity (%)	80.1 ± 6.5	29.0–101.2	50.8 ± 9.8

Kirindy Forest (CNFEREF) is a dry deciduous forest and is located further north (20.06714° S, 44.65745° E) (Kappeler and Fichtel 2012; Fig. 1a, e). The climate is also characterised by a hot wet season, during which ~ 96% of the annual rain falls (~ 900 mm), and a cold dry season that can last up to nine months with little to no precipitation (Kappeler and Fichtel 2012; Goodman et al. 2018). Compared to the southern spiny forest, Kirindy Forest is denser and has a closed canopy at 15–18 m (Fig. 1a, c). Importantly, no caves are known to be nearby and *M. commersoni* roosts solitarily in trees, exposed to highly variable environmental conditions (Reher and Dausmann 2021; Fig. 1b). Ambient conditions fluctuate greatly on a daily basis (Table 1), with highest *T*_a_ and lowest relative humidities (RH_a_) occurring in the early afternoon and lowest *T*_a_ and highest RH_a_ in the early morning. We did not trap any *M. commersoni* during the dry season, presumably because they were hibernating in tree hollows or had migrated to caves further away. Therefore, we are only able to present data from the wet season for Kirindy Forest.

To highlight the main structural differences between the two habitats that strongly affect the bats’ roost characteristics, we will henceforth refer to Tsimanampetsotse National Park as the “cave roost” and to Kirindy Forest as the “forest roost”.

### Trapping and handling

Cave bats were trapped in June/July 2016 and February/March 2017 (dry and wet seasons, respectively; Reher et al. 2018) and forest bats in February/March and July/August 2018 (wet and dry seasons, respectively; Reher and Dausmann 2021). We used different trapping methods owing to the differences in habitat structures and bats’ roosting behaviours. During the dry season, we hand-caught adult cave-dwelling bats in the early mornings between 07:00 and 09:00 h because this species is generally inactive at this time of year. During the wet season we erected a two-bank 4.2 m^2^ harp trap (Faunatech Austbat, Bairnsdale, Australia) in front of the same cave’s entrance. In the forest, two to three mist nets (3 m height × 6 m length, 19 mm mesh; Ecotone, Sopot, Poland) were opened each night in major flight corridors. Regardless of habitat, the harp trap and mist nets remained open for the first three hours after sunset and were checked every 10 to 20 min, depending on season. Per trapping event, we transferred the first two adult bats into a cloth bag while additional adults and juveniles were released immediately at the capture site. Only non-reproductive individuals were studied.

All captive bats were processed at the site of capture within 30 min. They were weighed, sex was determined and forearm length was measured. A patch of fur was removed from between the shoulder blades using a razor and shaving cream to allow the attachment of a temperature-sensitive radio transmitter (~ 0.9 g, Pip Ag376; Biotrack, Wareham, UK) using a medical skin glue (Osto-Bond, Montreal, Canada or Manfred Sauer GmbH, Lobbach, Germany). The thermal sensor was placed on the skin and after attachment the transmitter itself was partially covered by the bat’s fur. Transmitters weighed ≤ 2.6% of a bat’s body mass (mean = 1.8 ± 0.43%) and thus well below recommended maxima (e.g., Aldridge and Brigham 1988). Prior to attachment transmitters were calibrated in a water bath from 3–45 °C against a precision thermometer traceable to a national standard. Skin temperature (*T*_skin_) obtained via external transmitters provides a non-invasive and reliable proxy of *T*_b_, particularly in small mammals (Audet and Thomas 1996; Dausmann 2005; Langer and Fietz 2014; but see Willis and Brigham 2003). We marked all bats with an individual, three-digit wing tattoo using non-toxic ink (Hauptner-Herberholz, Solingen, Germany) after the membrane was locally anaesthetised (EMLA, AstraZeneca, Wedel, Germany). Animal handling lasted about 10 min and never exceeded 15 min.

### Respirometry

We measured metabolism as the rate of oxygen consumption ($\dot{V}$O_2_) using an open-flow respirometry system in pull mode. Directly after processing, bats were transferred into individual 2 L plastic metabolic chambers, which were equipped with a net for roosting. Each chamber had small holes as air inlets in one wall and an outlet was connected to a portable gas analyser on the opposite side (OxBox; T. Ruf and T. Paumann, University of Veterinary Medicine Vienna, Austria), which was powered by a standard 12 V car battery. During measurements, sample air was drawn from the metabolic chambers at a constant flow of 50 L h^−1^ using a diaphragm pump, then dried with silica gel and filtered before passing through the OxBox’s integrated mass flowmeter, and an aliquot thereof was drawn through the gas analyser. The oxygen content of the air was measured every 10 s for 55 min. For the remaining 5 min per hour, reference air from outside the metabolic chambers (also dried with silica gel and filtered) was analysed for oxygen content to control for drift in the electrochemical oxygen sensors (7OX-V CiTicel; City Technology, Portsmouth, UK). This was used to correct the sample air values with the software Clampfit v10.3.1.4 (Molecular Devices, Sunnyvale, USA). The oxygen sensors were calibrated in the laboratory before and after measurements using calibration gases mixed by a gas mixing pump (19.9, 20.3 and 21.0% O_2_ content; 2KM300/a, Wösthoff Messtechnik GmbH, Bochum, Germany).

The whole set-up consisting of chamber, OxBox, pump and car battery was placed within the cave or forest to measure the bats’ metabolism under natural ambient conditions. Within the cave, we placed the measurement set-up in an adjoining chamber next to the bats’ roosting chamber to avoid unnecessary disturbance of the whole colony. Although this chamber was slightly cooler by ~ 3.1 °C in the dry season and ~ 1.1 °C in the wet season, it was frequently visited by individual *M. commersoni*. Within the forest, we placed the measurement set-up in a shaded enclosure near the camp in the centre of the forest to avoid abundant local predators such as fossas *Cryptoprocta ferox*.

During each respirometry run we recorded *T*_a_ and RH_a_ using a data logger placed within each respirometry chamber (Hygrochron iButtons, Maxim integrated, San Jose, USA) and *T*_skin_ using a remote receiver/logger placed next to the setup (DataSika SRX-800-D; Biotrack, Wareham, United Kingdom). All temperature data were recorded at 5-min intervals.

We started measurements immediately after capture and processing, usually between 21:00 and 23:00 h in the wet season and 09:00 and 11:00 h in the dry season owing to different trapping times. We used the initial metabolic rate values after a bat was introduced to the metabolic chamber as indicators of individual stress levels. If there was no clear reduction in these first values within the next 30 or 60 min (depending on inactive and active phase, respectively), bats were released at their point of capture to avoid continuous stress. After the first 24 h, all individuals were provided with fresh water (1 ml) and food (~ 2 g), but only three individuals touched the food. The three hours after feeding were omitted from analyses (see Reher et al. 2018). One respirometry run typically lasted ~ 45 h, thus included the activity and rest phase, and ended with the beginning of the bats’ usual active phase between 17:30 and 18:30 h to ensure immediate foraging opportunities. Before the bats were released at their capture site, they were weighed and offered food and water. All applicable institutional and national guidelines for the care and use of animals were followed.

### Data processing and analysis

We analysed all data using Cran R (R Core Team 2018) in “RStudio” (RStudio Team 2016) and the packages “plyr” (Wickham 2011), “dplyr” (Wickham et al. 2020), “readxl” (Wickham and Bryan 2017), “lubridate” (Grolemund and Wickham 2011), “ggplot2” (Wickham 2016), “ggpubr” (Kassambara 2020a), "cowplot" (Wilke 2020). Data are shown as mean ± standard deviation and range if appropriate; *N* represents the number of individuals, *n* the number of included data points.

We first calculated the rate of oxygen consumption ($\dot{V}$O_2_) as ml O_2_ h^−1^ corrected to standard temperature and pressure, dry with Eq. 11.2 in Lighton (2008) before dividing by average body mass (BM) during a measurement to determine mass-specific metabolic rate (MR, ml $\dot{V}$O_2_ g^−1^ h^−1^). We assumed an average respiratory quotient of 0.85 (oxidation of 50% fat and 50% carbohydrate, Dausmann et al. 2009). We determined different physiological states via visual inspection of MR patterns following Reher and Dausmann (2021). We defined torpor as a decrease in MR by at least 50% compared to resting MR (RMR), which lies within the range of the highest metabolic reductions seen during torpor in warm environments (25–84%; Song et al. 1997; Dausmann et al. 2009; Grimpo et al. 2013; Kobbe et al. 2014). For downstream analysis, we removed arousal and entry phases and continued with a subset of MR data. We only used the lowest 50% of resting metabolic rate (RMR) values per hour per individual during the bats’ usual resting phase, i.e. from sunrise to 30 min before sunset, to ensure that data from any active or disturbed animals were excluded (Bethge et al. 2017; Reher and Dausmann 2021; Rodgers and Franklin 2021). For TMR, we included the lowest 70% of data per hour per individual. We differentiated between micro-torpor bouts, the mean duration of which were as short as 17 ± 8 min (range: 5–36 min, *n* = 857; mTMR), and extended torpor bouts lasting 4.9 h ± 58 min (range: 1.7–8.3 h, *n* = 44; eTMR) after Reher and Dausmann (2021). Briefly, micro-torpor bouts have a similar reduction in MR to extended torpor bouts but bats alternate rapidly between torpid and euthermic states without variation in *T*_skin_, whereas extended bouts have characteristically slower entry and arousal curves and a clear deviation in *T*_skin_ from euthermia. We acknowledge that brief reductions in MR could also be related to a reduced respiratory rate or periods of apnoea interrupted by short breathing bursts. These are regularly observed during hibernation (e.g., Thomas et al. 1990) or shorter torpor bouts at low *T*_a_ (e.g., Morris et al. 1994), but so far not during torpor at *T*_a_ > 25 °C (Levin et al. 2015; Geiser 2021). Furthermore, MR drops of ~ 10% are common during sleep (Heller 1987), although the reduction might amount to ~ 35% depending on sleep states (slow-wave sleep and rapid-eye-movement sleep) and *T*_a_ (Glotzbach and Heller 1976; Rechtschaffen 1998; Cooper and Withers 2002). However, since we observed reductions of ~ 76% (range: 63–89%) and at high *T*_a_ (22–36 °C), we are confident that the bats were indeed torpid.

Overall reductions in MR during torpid states were compared among roosting conditions using *t* tests; *p* values were adjusted with Bonferroni-Holm corrections to account for multiple comparisons (package “rstatix”; Kassambara 2020b).

*T*_skin_ during the respirometry runs was calculated using second-order polynomial regressions obtained from the calibration curves (all *R*^2^ ≥ 0.99) and used as a proxy for *T*_b_. Deviations in torpid *T*_skin_ from euthermia were analysed using paired-samples *t* tests with *p* values adjusted with Bonferroni-Holm corrections.

#### Bat morphology

To compare body mass, forearm length, body condition (standardized mass index, SMI, following Peig and Green 2009, 2010) and TMR among the different roosting conditions we used *t* tests adjusted for unequal variances if necessary or Wilcoxon signed-rank tests (package “rstatix”). In all cases, *p* values were adjusted with Bonferroni-Holm corrections.

#### Torpor bout timing and occurrence

We used Rayleigh’s tests and Watson two-tailed tests to determine whether the timing of torpor entry and arousal differed significantly from a random distribution and to identify differences in timing between sites and seasons (package “circular,” Jammalamadaka and Sengupta 2001). Time is given as circular mean ± standard deviation.

Torpor occurrence was compared between sites and seasons using two-sided Fisher’s exact tests; *p* values were adjusted with a Bonferroni-Holm correction (package “rstatix”).

#### Metabolic rates

To analyse MR, we allocated RMR and TMR to different *T*_a_ bins by rounding *T*_a_ to the nearest integer and assigned individual means of the different metabolic states to each *T*_a_ step to avoid pseudo-replication. We then explored the influence of roost site and season (as site-season: cave dry season, cave wet season, forest wet season), sex, body condition and roost temperature on metabolic rate using either linear mixed models (LMEs) or generalized linear mixed models (GLMMs; package “lme4”, Bates et al. 2015). We did not include RH_a_ in the models because *T*_a_ and RH_a_ were strongly negatively correlated (*R*^2^ = − 0.78) but we report RH_a_ mean and range during the measurements. Only *T*_a_ steps at which we measured at least three bats per roosting condition were included. We first fitted separate models for extended torpor MR (eTMR), micro-torpor MR (mTMR) and RMR, in which site-season, *T*_a_, the interaction of site-season and *T*_a_, sex and body condition were defined as fixed factors and bat ID was a random effect to account for repeated measures. Because MR is strongly associated with *T*_a_, which was almost constant in the cave but fluctuated considerably in the forest, we second modelled RMR and mTMR as a function of the roosting site at only overlapping temperature bins (28–30 °C and 31–34 °C) with bat ID as random effect (the site-season variable in these models contains only two levels respectively, as the *T*_a_ in the cave did not overlap seasonally). For analysing the subset of eTMR, we used *t* tests owing to the small sample size of bats measured at overlapping *T*_a_ steps (N = 3 bats per site and *T*_a_) and non-replicated data. We excluded data from one bat that entered a single multi-day torpor bout (> 3 days) for the analysis of eTMR and the subsequent analysis of torpor bout duration (see below). Please note that we could not directly compare the cave-dwelling bats’ MR between seasons because of no shared *T*_a_ steps (Fig. 4a–c, left panel).

Data exploration and validation was achieved following Zuur et al. (2010) and Zuur and Ieno (2016). Significance was calculated using Satterthwaite’s method (package “lmerTest”; Kuznetsova et al. 2017).

#### Torpor bout duration and frequency

We analysed the effect of sex, body condition and site-season on micro-torpor bout frequency, micro-torpor bout duration and extended torpor bout duration using separate GLMMs (package “glmmTMB”; Brooks et al. 2017). Micro-torpor bouts occurred multiple times each day so an average bout frequency per day was used in analyses. We did not model extended torpor bout frequency because there was rarely more than one bout per day and instead report proportions of bats torpid within the populations. In all models, we included site-season, sex and body condition as fixed factors and bat ID as a random effect. For extended torpor bout duration, we defined an additional model including the interaction of site-season and body condition, and compared both using likelihood ratio testing (Zuur et al. 2009). No environmental predictor (i.e., *T*_a_, RH_a_) was included because the site-season term encompassed per day differences in environmental variation. We ran pairwise comparisons between sites and seasons using Tukey’s HSD test adjusted for multiple comparisons based on estimated marginal means or, in case the model included a significant interaction term, estimated marginal slopes (package “emmeans”; Lenth 2021).

#### Resting energy expenditure

To evaluate whether there were differences in bats’ energetic costs among roosting conditions, we calculated individuals’ daytime resting energy expenditure (DREE, in kJ) between sunrise and sunset from per-minute metabolic rate values using an oxycalorific equivalent of 20.37 kJ/L O_2_, derived from an assumed respiratory quotient of 0.85 (Schmidt-Nielsen 1997). This approach takes into account different daytime torpor strategies, e.g., remaining euthermic (*n* = 5), entering only micro-torpor (*n* = 42), entering only extended torpor (6.9 h ± 59 min *N* = 10) or entering a combination of micro-torpor with a shorter extended torpor bout (4.4 h ± 54 min; *N* = 35). We analysed the effects of sites and seasons on DREE using a GLMM; site-season, sex, body condition and torpor strategy were included as fixed factors and bat ID as a random effect. Pairwise comparisons between sites and seasons were performed using Tukey’s HSD test based on estimated marginal means (see above).

## Results

We measured the physiological responses of 41 individual *M. commersoni*; 25 from the cave roost and 16 from the forest roost (Table 2). We studied cave bats during the wet and dry season. In the forest, however, we did not trap any bats during the dry season despite 261 trapping hours and thus only present forest data from the wet season (Table 2). Bats from the forest population were larger but not heavier than their conspecifics from the cave roost (Table 2, Table S1). Interestingly, body condition was similar in both roosting conditions and sexes, but the forest population’s females had a significantly lower body condition than all other bats (*t* test, *t*_27.7_ = − 6.54, *p* < 0.001; Table 2).

**Table 2:** Overview of key physiological and morphological variables of the different populations in the dry and wet seasons

	Cave roost				Forest roost	
	Dry season		Wet season		Wet season	
(a)	Female (6)	Male (4)	Female (9)	Male (6)	Female (9)	Male (7)
Body mass (g)°	45.2 ± 6.8^a^	55.5 ± 12.7^a^	45.1 ± 7.9^a^	53.8 ± 10.4^a^	46.3 ± 6.8^a^	79.6 ± 8.0^a^
Forearm length (mm)°	79.2 ± 1.8^a^	85.5 ± 1.9^a^	79.5 ± 1.0^a^	85.8 ± 2.1^a^	88.0 ± 1.4^b^	94.4 ± 2.8^b^
Body condition (SMI)°	60.9 ± 10.7^a^	54.9 ± 9.6^a^	59.6 ± 12.2^a^	53.1 ± 10.8^a^	41.0 ± 4.9^b^	53.8 ± 4.6^a^
Bats that used extended torpor	*N* = 3	*N* = 3	*N* = 1	*N* = 5	*N* = 8	*N* = 7
Bats that used micro-torpor	*N* = 5	*N* = 3	*N* = 8	*N* = 6	*N* = 9	*N* = 7

(b)	Dry season		Wet season		Wet season	
MR during rest (ml h^−1^ g^−1^)	1.04 ± 0.19		1.09 ± 0.36		1.05 ± 0.42	
MR during micro-torpor (ml h^−1^ g^−1^) (*reduction [%]*)	0.22 ± 0.05 (78.8 ± 4.8^*a*^)		0.30 ± 0.07 (72.9 ± 11.8^*a*^)		0.24 ± 0.07 (77.5 ± 7.4^*a*^)	
MR during extended torpor (ml h^−1^ g^−1^) (*reduction [%]*)	0.10 ± 0.02 (89.4 ± 0.8^*a*^)		0.17 ± 0.04 (82.4 ± 7.1^*b*^)		0.20 ± 0.05 (80.2 ± 5.3^*b*^)	
*T*_skin_ during euthermia (°C)^∆^	36.4 ± 1.7^*a*^		36.8 ± 1.5^*a*^		36.6 ± 2.0^*a*^	
*T*_skin_ during micro-torpor (°C)^∆^	36.1 ± 1.4^*a*^		35.9 ± 1.2^*a*^		36.1 ± 1.9^*a*^	
*T*_skin_ during extended torpor (°C)^∆^	33.5 ± 2.9^*a*^		33.0 ± 1.6^*a*^		38.5 ± 2.5^*b*^	
Extended torpor bout duration (min)	345.2 ± 190.4^a^		176.0 ± 83.6^b^		312.5 ± 157.1^a^	
Micro-torpor bout duration (min)	21.1 ± 16.9^a^		12.8 ± 8.9^b^		13.3 ± 6.8^b^	
DREE (kJ day^−1^ g^−1^)	0.10 ± 0.04^a^		0.18 ± 0.08^b^		0.11 ± 0.05^a^	

(a) The number of females and males studied are given in parentheses. Mean body mass, forearm length and body condition; the number of bats that entered either extended or micro-torpor; mean metabolic rate during extended torpor and micro-torpor and as a percentage reduction of resting metabolism; mean torpor bout duration for both modes of torpor; and daytime resting energy expenditure (DREE) are shown. (b) We pooled sexes when calculating means of all physiological variables because sex was not included in the most parsimonious models. For simplicity, MR reduction was compared over the mean of the entire measured *T*_a_ range because testing among subset *T*_a_ ranges yielded the same trends. Different superscript letters indicate statistical differences (see “Results” for details)

°only within sex comparisons across roosting conditions (see supplements)

^∆^only analysed within sites and seasons, i.e. letters indicate differences observed among metabolic states under the same roosting condition

### Occurrence of torpor and deviations from euthermia

**Fig. 2:**
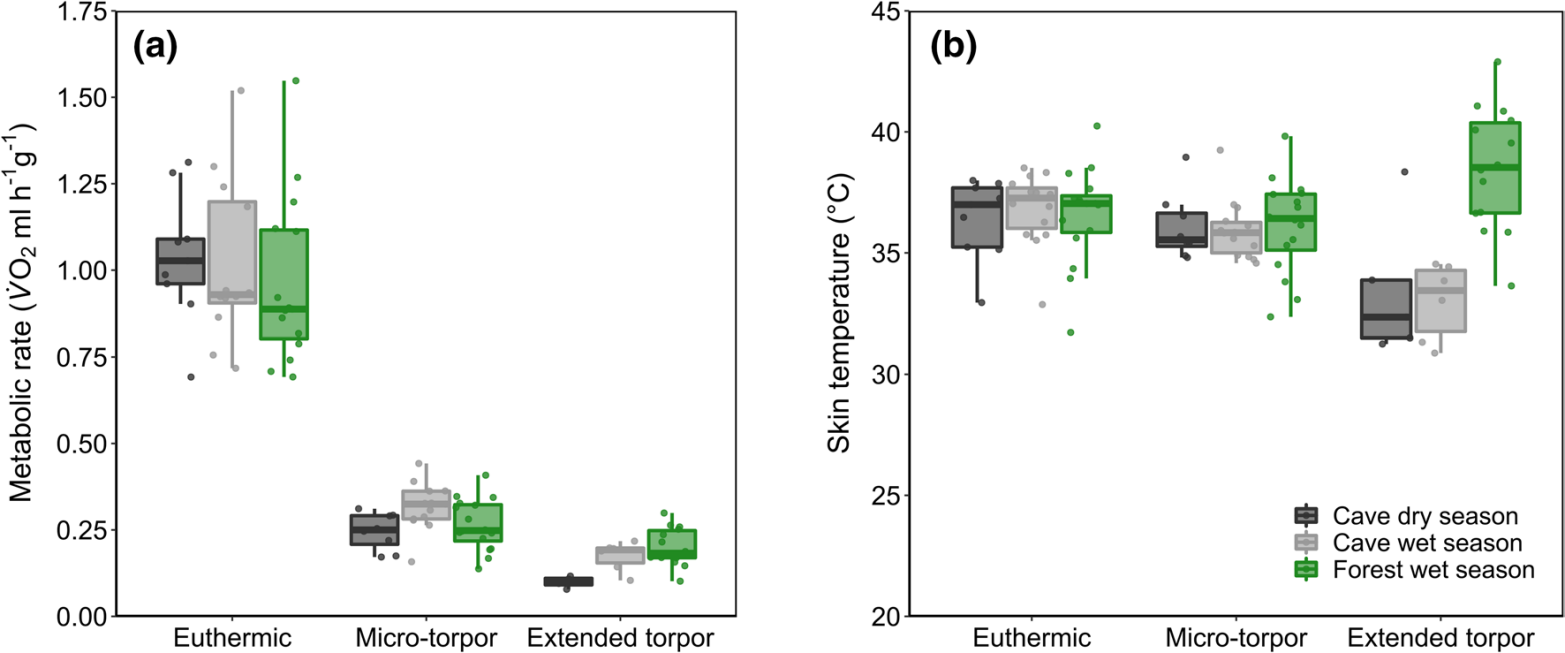
**a** Mean mass-specific metabolic rate ($\dot{V}$O_2_ (MR, ml h^1^ g^1^) and **b** skin temperature (*T*_skin_, °C) of animals at rest (euthermic), in a micro-torpor bout and in an extended torpor bout. Bats roosting in a cave during the dry (dark grey) and wet season (light grey) and forest-dwelling bats during wet season (green). The centre line represents the median, box limits indicate upper and lower quartiles, whiskers show 1.5 × interquartile range and points are all included data

Most bats (38 out of 41) entered torpor (*n* = 44 extended bouts, *n* = 857 micro-torpor bouts). Only three cave-roosting individuals remained normothermic over the whole measurement period (one female in the wet season and one female and one male in the dry season). During the wet season the forest bats made extensive use of both modes of torpor (94% used extended torpor and 100% used micro-torpor), and more bats used extended torpor compared to the cave population (Fisher’s exact test, *p* = 0.006), which opted for micro-torpor (94%) rather than extended torpor (40%; Fisher’s exact test, *p* = 0.005). During the dry season, a comparable proportion of bats used extended torpor (60%; Fisher’s exact test, *p* = 0.428) and micro-torpor (80%; Fisher’s exact test, *p* = 0.968). Overall mTMR was reduced by ~ 75% compared to RMR, without differences between sites or seasons (cave dry vs. cave wet season: *t*_21.0_ = 1.13, *p* = 0.271; cave dry vs. forest wet season: *t*_17.9_ = − 1.62, *p* = 0.246; cave wet vs. forest wet season: *t*_21.8_ = − 2.54, *p* = 0.057; Table 2). The greatest reductions in eTMR were observed in the cave during the dry season (89.4 ± 0.8%; cave dry vs. cave wet season: *t*_9.25_ = − 2.51, *p* = 0.031; cave dry vs. forest wet season: *t*_11.2_ = − 3.30, *p* = 0.004) and comparable reductions in the wet season regardless of roost type (forest: 80.2 ± 5.3%, cave: 82.4 ± 7.1%; *t*_8.51_ = − 1.262, *p* = 0.223; Fig. 2a). Although the drop in MR during torpor was considerable, changes in *T*_skin_ were less evident (Table 2, Fig. 2a, b): it was not possible to detect micro-torpor bouts through a clear *T*_skin_ signal under any roosting conditions studied (cave dry season: *t*_7_ = 0.14, *p* = 0.185; cave wet season: *t*_13_ = 1.73, *p* = 0.071; forest wet season: *t*_15_ = 0.86, *p* = 0.402). However, *T*_skin_ deviation from euthermia indicated an extended torpor bout. In the cave, *T*_skin_ dropped by 2.9 ± 2.0 °C during extended torpor (cave dry season: *t*_4_ = 3.29, *p* = 0.047; cave wet season: *t*_5_ = 4.55, *p* = 0.014) but increased by 1.9 ± 2.8 °C in the forest (*t*_13_ = 3.18, *p* = 0.022; Table 2, Fig. 2b), where extended torpor was most commonly associated with higher *T*_a_ than in the cave (Reher and Dausmann 2021).

### Temporal synchronisation of torpor bouts

**Fig. 3:**
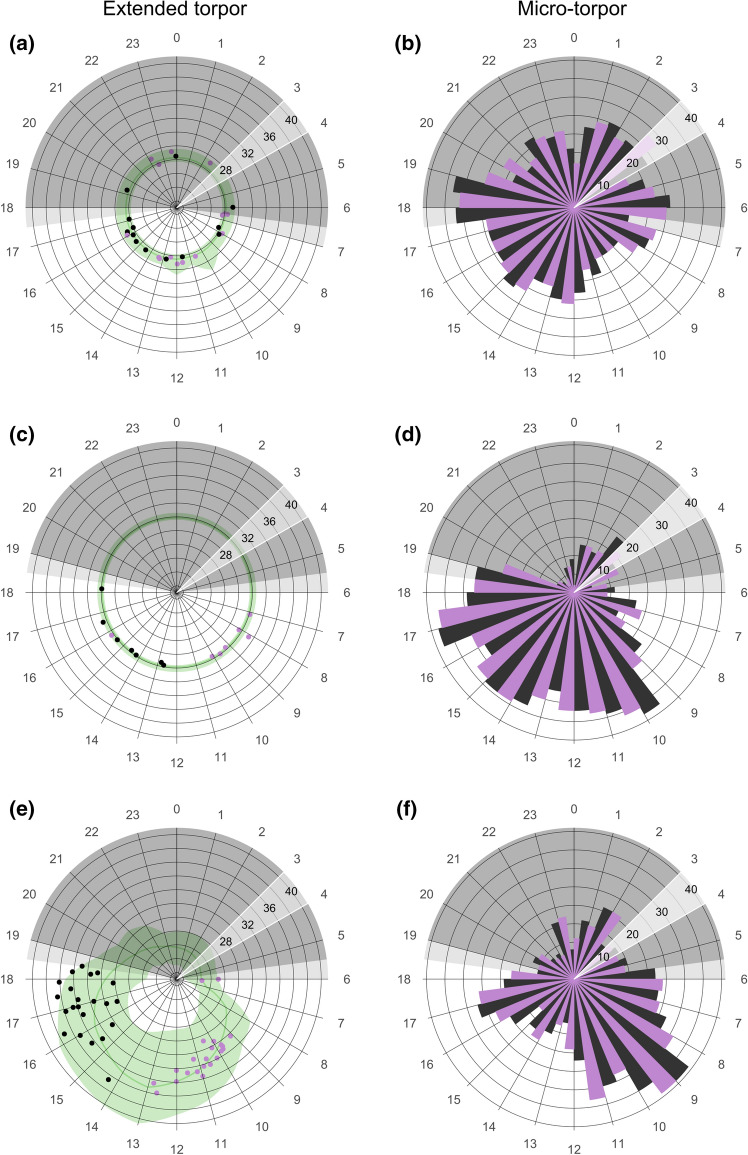
Timing of entry into (mauve), and arousal from (black), extended and micro-torpor bouts under three different roosting conditions: in a cave during the dry and wet seasons (**a**, **b** and **c**, **d**, respectively) and in a forest during the wet season (**e**, **f**). For extended torpor (**a, c, e**), the data show the time of the day (circular axis) and skin temperature (°C; radial axis distance). Dark grey shaded areas indicate scotophase and light grey areas indicate twilight. The green line illustrates hourly mean ambient temperature with hourly minima and maxima bounding the green shaded areas. For micro-torpor (**b**, **d**,** f**), the data show the occurrence of micro-bout entries (mauve) and arousals (black; radial distance) over the course of the day at hourly intervals

We found seasonal and site-specific timing in entry into torpor and arousal from torpor. The entry and arousal times of both extended and micro-torpor bouts were randomly distributed in the cave during the dry season (table S2, Fig. 3a, b), when the bats did not leave the cave for months. In the wet season, extended torpor and micro-torpor entry and arousal followed a clear pattern at both sites (Table S2, Fig. 3c, d, e, f), and the timing of extended torpor entry and arousal was more synchronized than for micro-torpor (i.e., extended torpor bout times had higher Rayleigh’s r-values). The bats in the cave entered into and aroused from extended torpor earlier than in the forest (entry: *x* = 0.24, *p* < 0.05, cave 09:21 ± 40 min, forest 10:23 ± 12 min; arousal: *x* = 0.1872, *p* < 0.05 cave 14:54 ± 29 min, forest 16:56 ± 17 min; Table S2; Fig. 3c, e). Micro-torpor bouts appeared to occur around the clock in the wet season but entry and arousal times differed from a random distribution (Table S2, Fig. 3). In the cave, both were more frequent during the day (entry: 12:58 ± 5 min, arousal: 13:18 ± 7 min), whereas in the forest entries and arousals occurred predominantly in the mornings (entry: 09:28 ± 9 min, arousal: 09:43 ± 11 min; Table S2; Fig. 3d, f), often before extended torpor bouts at high *T*_a_.

### The effect of roosting environment on MR

**Fig. 4:**
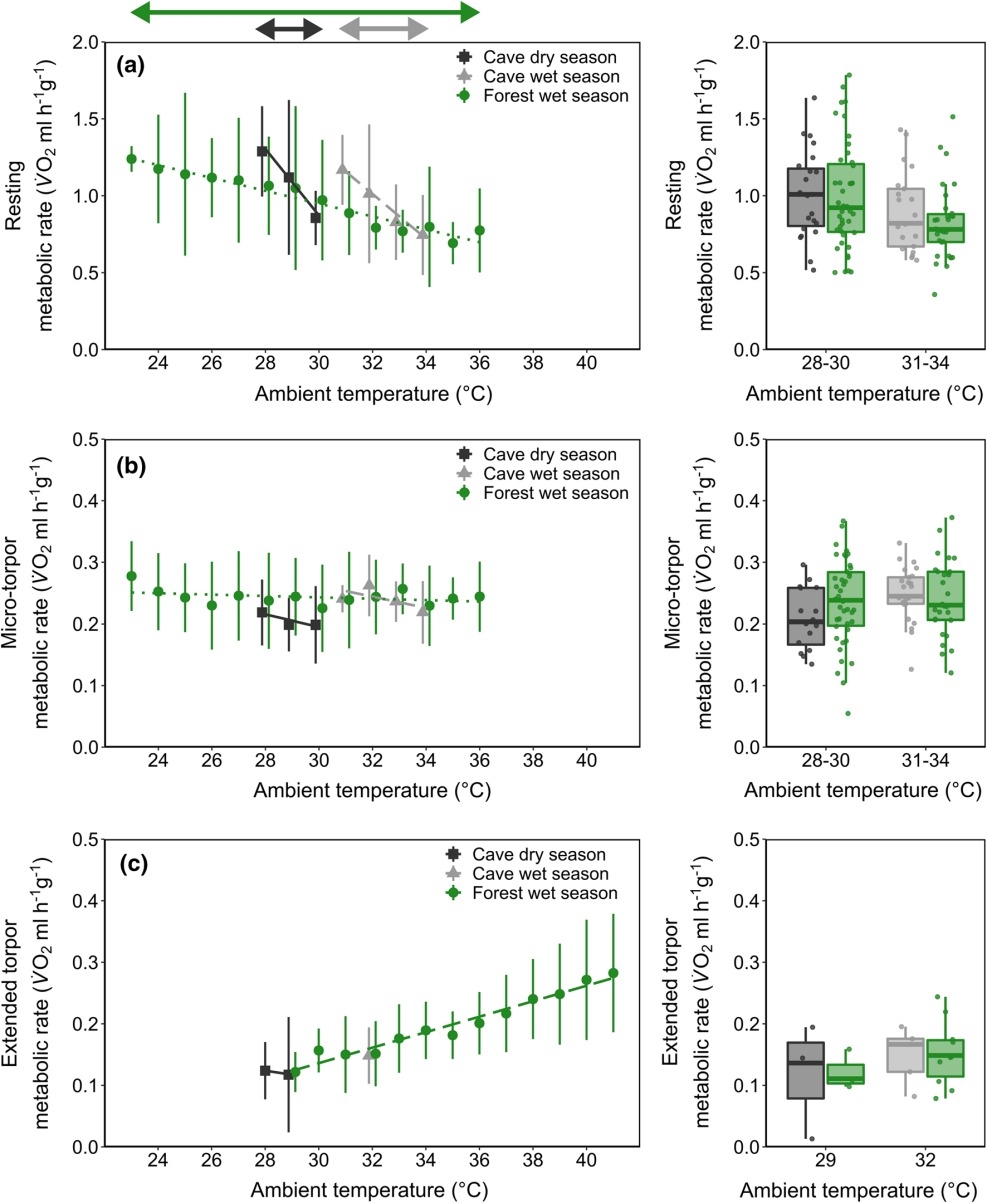
The mass-specific metabolic rate (MR, ml $\dot{V}$O_2_ h^−1^ g^−1^) of bats when **a** resting, **b** in micro-torpor and **c** in extended torpor in a cave in the dry season (dark grey squares), the same cave in the wet season (light grey triangles) and a tree roost in the wet season (green circles). The left panel shows MR as a function of ambient temperature; error bars represent standard deviation. The arrows above the left panel indicate the *T*_a_ range and colours correspond with roost/season. The right panel shows MR at only overlapping ambient temperature (centre line, median; box limits, upper and lower quartiles; whiskers, 1.5 × interquartile range; dots, data included). Please note the different *y*-axis scale of **b** and **c**

We analysed the RMR of 39 individuals. The interaction of site-season and *T*_a_ step (*Chi*^*2*^ = 8.70, *p* = 0.013) had a significant effect on RMR. RMR generally decreased with increasing *T*_a_ (*t* = − 3.30, *p* < 0.001), but the slope was significantly flatter in the forest (forest wet vs. cave dry season: *z-ratio* = 2.64, *p* = 0.023; forest wet vs. cave dry season: *z-ratio* = − 1.97, *p* = 0.036; Fig. 4a, left panel) than in the cave, where the slope did not differ between seasons (*z-ratio* = -0.92; *p* = 0. 625; Fig. 4a, left panel). There was no significant variation in a subset of RMR at similar temperature ranges (28–30 °C: *t* = -0.50, *p* = 0.613, *N* = 26; 31–34 °C: *t* = − 0.45, *p* = 0.650, *N* = 25; Fig. 4a, right panel). Higher body condition was correlated with lower RMR (*t* = − 2.17; *p* = 0.030) but sex was not a significant predictor (*t* = 0.82; *p* = 0.412).

Micro-torpor MR decreased slightly with increasing *T*_a_ (*t* = − 4.52, *p* = 0.034, *N* = 38; Fig. 4b, left panel). Neither site-season (cave wet season *t* = 0.92, *p* = 0.362; forest wet season *t* = 0.42, *p* = 0.664), body condition (*t* = − 0.97, *p* = 0.340), nor sex significantly affected mTMR (*t* = 0.17, *p* = 0.870). Accordingly, site-season was not a significant predictor of a subset of mTMR at only similar temperature ranges (28–30 °C: *t* = 0.807, *p* = 0.43, *N* = 23; 31–34 °C: *t* = -0.97, *p* = 0.340, *N* = 26; Fig. 4b, right panel).

We analysed eTMR for 25 individuals. In the forest population, eTMR increased with increasing *T*_a_ (*t* = 8.87; *p* < 0.001; Fig. 4c, left panel) while sex (*t* = − 0.06; *p* = 0.951) and body condition had no significant effect (*t* = 0.11; *p* = 0.912). We found no differences in a subset of eTMR at similar *T*_a_ (29 °C: *t*_2.48_ = − 0.08, *p* = 0.946, *N* = 6; 32 °C: *t*_9.28_ = − 0.12; *p* = 0.909; *N* = 15; Fig. 4c, right panel).

### Torpor bout frequency and duration

**Fig. 5:**
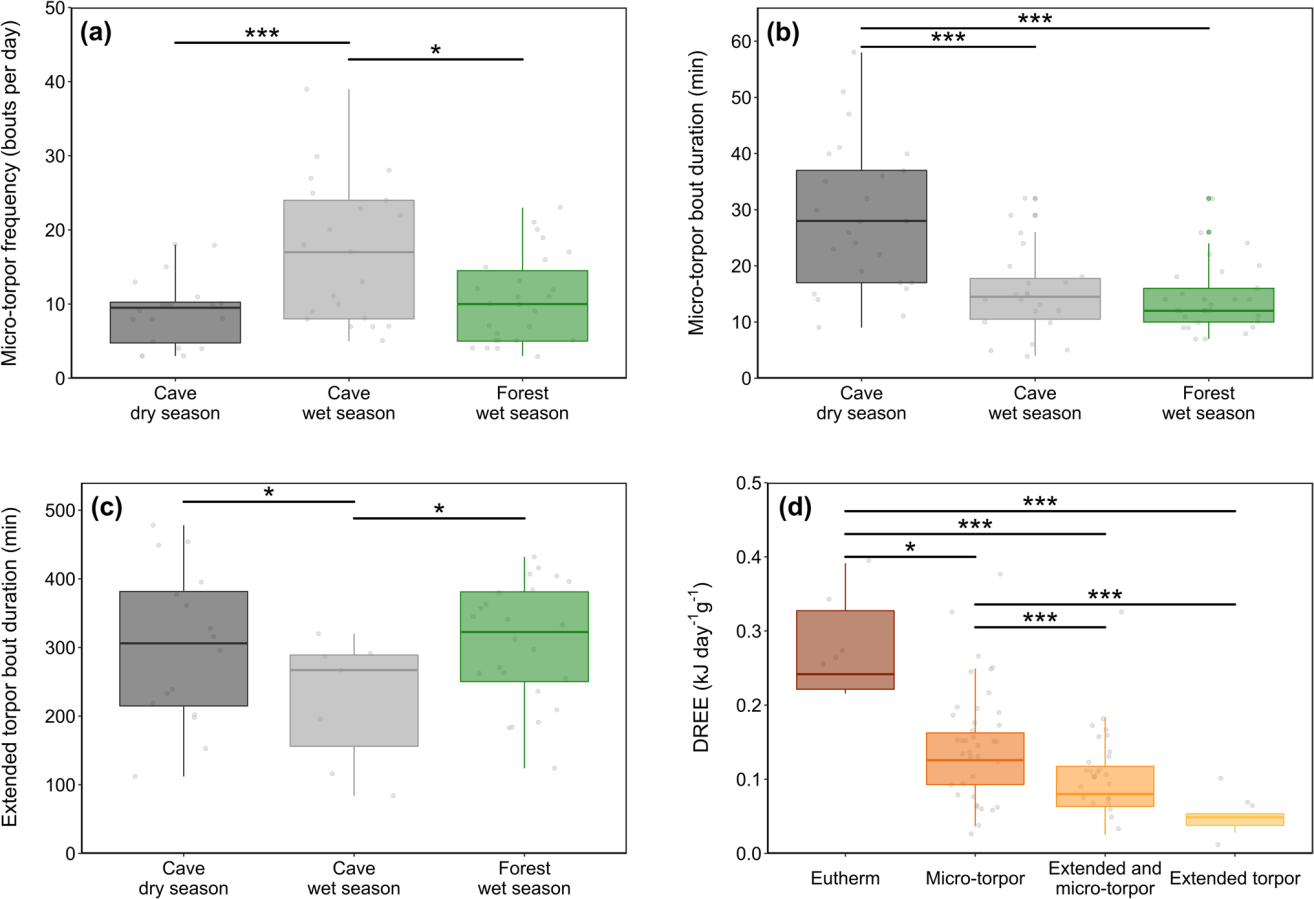
**a** Micro-torpor bout frequency, **b** micro-torpor bout duration and **c** extended torpor bout duration of individuals roosting in the cave in the dry season (dark grey) or wet season (light grey) and in the forest in the wet season (green). **d** shows daytime resting energy expenditure according to metabolic strategy (remaining euthermic, red; entering micro-torpor bouts, orange; entering micro-torpor bouts together with a more extended torpor bout, light orange; entering extended torpor, yellow). In all plots, the centre line represents the median, box limits indicate upper and lower quartiles, whiskers show 1.5 × interquartile range and grey points are all data included; significant differences are marked with asterisks (*p ≤ 0.05; **p < 0.01; ***p < 0.001)

Site-season affected micro-torpor bout frequency (*Chi*^*2*^ = 15.12, *p* < 0.001, *N* = 37, *n* = 76). The cave-dwelling bats in the wet season entered micro-torpor bouts more frequently than their conspecifics (cave wet vs. cave dry season: *z-ratio* = − 3.67, *p* < 0.001; cave wet vs. forest wet season: *z-ratio* = − 2.51, *p* = 0.033), while the frequency of micro-torpor bouts per day was similar for cave bats in the dry season and forest-dwelling bats in the wet season (*z-ratio* = − 1.33, *p* = 0.376; Fig. 5a). Micro-torpor bout frequency was influenced by sex, with females entering micro-torpor more frequently than males (*z* = − 2.27, *p* = 0.023); body condition had no effect on the frequency of micro-torpor bouts (*z* = 1.77, *p* = 0.077). For micro-torpor bout duration, site-season was the single significant predictor (*Chi *^*2*^= 22.37, *p* < 0.001, N = 37, *n* = 76). Cave-dwelling bats entered longer bouts in the dry season than bats in the wet season, regardless of roost type (cave dry vs. cave wet season: *z-ratio* = 4.18, *p* < 0.001; cave dry vs. forest wet season: *z-*ratio = 4.27, *p* < 0.001; cave wet vs. forest wet season: *z-ratio* = 0.26, *p* = 0.962; Fig. 5b). Neither sex (*z* = 0.16, *p* = 0.875) nor body condition (*z* = − 0.50, *p* = 0.617) influenced micro-torpor bout duration significantly.

Extended torpor bout duration was better explained when the interaction of site-season and body condition was included (*Chi *^*2*^= 3.97, *p* = 0.036). In cave conditions, extended torpor bout duration was negatively related to body condition but the slope of this relationship was steepest during the dry season than during the wet season (*t-ratio* = − 2.06, *p* = 0.013, *N* = 24, *n* = 48; figure S1). The duration of extended torpor bouts was shortest in wet season cave bats (cave wet vs. cave dry season: *t-ratio* = 2.93, *p* = 0.024; cave wet vs. forest wet season *t-ratio* = 2.34, *p* = 0.048) and similar between wet season forest bats and dry season cave bats (*t-ratio* = − 0.86, *p* = 0.655; Fig. 5c). We did not analyse the frequency of extended torpor bouts because the bats usually only entered one extended bout per day. Under all three different environmental conditions, the ratio of males entering extended torpor was always higher than females (cave dry season: females 50%, males 75%; cave wet season: females 11%, males 83%; forest wet season: females 89%, males 100%; Table 2).

### Daytime resting energy expenditure (DREE)

The physiological strategy used by the bats was the only significant predictor for total energy expenditure during daytime rest (DREE; *Chi*^*2*^ = 38.12, *p* < 0.001, *N* = 39, *n* = 83), i.e. either remaining euthermic (0.26 ± 0.10 kJ day^−1^ g^−1^), entering micro-torpor bouts (0.14 ± 0.06 kJ day^−1^ g^−1^), entering micro-torpor bouts together with an extended torpor bout (0.09 ± 0.05 kJ day^−1^ g^−1^) or only entering extended torpor (0.05 ± 0.03 kJ day^−1^ g^−1^; Fig. 5d). In this order, each response saved approximately 46% (*z-ratio* = 2.62, *p* < 0.043), 65% (*z-ratio* = 3.83, *p* < 0.001) and 81% (*z-ratio* = 4.01, *p* < 0.001) of the energy expended during a day remaining euthermic (Fig. 5d). Sex (*t* = 0.73, *p* = 0.468), body condition (*t* = − 1.05, *p* = 0.294) and site-season (*Chi *^*2*^= 5.77, *p* = 0.056) had no effect on DREE. However, the cave population in the wet season had a higher DREE than the other bats (cave wet vs. cave dry season: *z-ratio* = -17.63, *p* < 0.001; cave wet vs. forest wet season: *z-ratio* = − 10.80, *p* < 0.001; cave dry vs. forest wet season: *z-ratio* = − 1.43, *p* = 0.094; Table 2).

## Discussion

The two populations of *M. commersoni* studied showed variation in their physiological responses to environmental conditions experienced while roosting in contrasting habitat types. For both populations, torpor was a key response used to finely balance energy expenditure. While metabolic rate during rest and during torpor were both similar across roosts, torpor timing, duration and frequency were flexibly adjusted to prevailing *T*_a_ and RH_a_.

Surprisingly, cave-dwelling bats used relatively low rates of torpor in the resource-poor and cooler dry season. During this time of the year, the bats rarely leave their cave for months and food availability is drastically reduced (Razakarivony et al. 2005; Goodman 2006; Rakotoarivelo et al. 2007; Reher et al. 2019). Hence, we hypothesised that bats roosting in the cave would hibernate and show prolonged torpor (i.e., torpor bouts > 24 h in duration) most often. Instead, only 60% of these individuals entered extended torpor and 80% used repetitive micro-torpor bouts, which was less than during the warmer, more abundant wet season (93%) or site (100%). Only one measured individual entered a multi-day torpor bout and it appears to be common that several strategies, i.e. hibernation (multi-day torpor), extended torpor, micro-torpor and no torpor, are used by different individuals within the same population in this species (Reher et al. 2018; Dausmann et al. in press). This variability was also observed in the Malagasy mouse lemur *Microcebus griseorufus* (Kobbe et al. 2011). Interestingly, the timing of both extended and micro-torpor occurred randomly throughout the day unlike during the wet season, when bats left the cave at dusk. Photoperiod is often a stimulus for torpor timing but in the absence of light *T*_a_ can also act as an important *Zeitgeber* (Heldmaier et al. 1982; Körtner and Geiser 2000). In Andranolovy Cave, however, *T*_a_ and RH_a_ were relatively constant. Animals living in a constant environment like this can experience shifts in their circadian rhythm. For instance, patterns of activity become arrhythmic in arctic mammals when they are confronted with continuous dark or light conditions (van Oort et al. 2005; Appenroth et al. 2021) and circadian patterns in the timing of arousal from torpor are lost in some hibernators (e.g., Körtner and Geiser 2000; Revel et al. 2007; Williams et al. 2017). The loss of circadian rhythmicity in torpor timing therefore supports the notion that these bats cease foraging during this time of year (Razakarivony et al. 2005; Goodman 2006; Rakotoarivelo et al. 2007; Reher et al. 2019), even though it is likely that not all were hibernating. We suggest that a stable microclimate within the bat’s thermal neutral zone (32–36 °C; Reher and Dausmann 2021; this study), i.e. the *T*_a_-range at which no active thermoregulatory support is needed to maintain euthermic *T*_b_, permits an overwintering strategy fuelled by large fat deposits without the need for long-term hypometabolism.

Ample food resources are available in the wet season, making longer torpor bouts unnecessary. Less time spent torpid, and thus longer active periods, benefit social interaction in a large colony (Kunz and Lumsden 2003). The cave-dwelling bats we studied used fewer extended torpor bouts in the wet season (40% of individuals) and these bouts were shorter than during the dry season or in the forest. Three out of six animals using extended torpor in the cave were measured when the outer fringes of a cyclone crossed the region and the colony did not leave the cave for several days. Torpor is a powerful response for enduring extreme weather events such as droughts, heatwaves, storms and fires (Doucette et al. 2012; Bondarenco et al. 2014; Nowack et al. 2015; Stawski et al. 2015). Moreover, it can also be advantageous in better-resourced seasons (Heldmaier et al. 2004; Geiser and Brigham 2012); for example, short torpor bouts can compensate for unsuccessful foraging (Heldmaier et al. 2004) or speed up pre-hibernation fattening (Giroud et al. 2012). Because *M. commersoni* accumulates fat at the end of the wet season (Goodman 2006; Jenkins and Racey 2008), extended torpor may be used flexibly on an individual basis or to get through environmental bottlenecks.

In contrast, forest-roosting bats made considerable use of torpor in the wet season (94% of individuals), which was more than the cave bats regardless of season (60% of individuals in the dry season and 40% in the wet season). Furthermore, extended torpor bouts used by forest bats in the wet season were as long as those used by dry season cave bats. This was unexpected because the foliage used for roosting did not offer protection from predators or daytime extremes of high *T*_a_ or low RH_a_. Water loss is presumably high under these conditions, as insensible water loss increases as ambient water vapour pressure decreases (Mitchell et al. 2018; but see Cooper and Withers 2017) and the maximum difference in RH_a_ between the cave and forest was 65% (cave: never below 94% RH_a_, forest: down to 29% RH_a_ after noon). During torpor water loss can be reduced by over 90% compared to euthermia, because water-consuming processes such as respiration, urine production and defecation are downregulated or even stopped (Morris et al. 1994; Webb et al. 1995; Muñoz-Garcia et al. 2012; Levin et al. 2015; Hill et al. 2016). Indeed, the timing of longer torpor bouts in *M. commersoni* is related to times when *T*_a_ exceeds euthermic *T*_b_ (Reher and Dausmann 2021). Above this threshold, only evaporative cooling can regulate *T*_b_, which is unfavourable in a dry region if water reserves are unable to be replenished (Mitchell et al. 2018). Entering torpor at high *T*_a_ reduces metabolic heat and water production, allowing higher rates of heat from the environment to be stored in the body via facultative hyperthermia (Lovegrove et al. 2014; Welman et al. 2017; Reher and Dausmann 2021). Compared to the bats roosting in the cave at near stable conditions, it appears the forest bats in our study used this strategy to mitigate heat and water stress.

In addition to modulating torpor frequency and duration, heterotherms can adjust the level of metabolic reduction to reduce the costs of torpor while maximising the benefits (Boyles et al. 2020). The mean reduction in eTMR from euthermia (80–89%) and mTMR (73–79%) was relatively similar among the different roosting conditions. Given that *T*_skin_ during torpor ranged between 33.0 °C in the cave and 38.5 °C in the forest (total range: 24.6–42.9 °C), this reduction in MR is remarkable and among the highest reported for warm environments (25–84%; Song et al. 1997; Dausmann et al. 2009; Grimpo et al. 2013; Kobbe et al. 2014). The observed variability in metabolic depression reflects the *T*_a_ fluctuations at each roost. Bats were torpid at a higher *T*_a_, and therefore *T*_b_, in the forest than in the cave. The forest and cave bats had a similar TMR at the same *T*_a_, comparable to Kuhl's pipistrelle and big brown bats that also display a TMR that varies very little between populations roosting under different climatic conditions (Klüg-Baerwald and Brigham 2017; Gearhart et al. 2020). Therefore, the level of metabolic depression at a given *T*_a_ appears to be less plastic than torpor pattern variation in these bats (but see Dunbar and Brigham 2010). It is worth noting, however, that we excluded one individual that entered a multi-day torpor bout from analyses. The body condition of this individual was among the poorest 20% of bats in this study and it reduced MR by 94% at a *T*_skin_ of 33 °C. TMR during multi-day torpor is lower than during daily torpor (Geiser 2004) and it is possible that this individual entered a multi-day torpor bout to maximise energetic savings and slow the depletion of its meagre fat reserves (Humphries et al. 2003; Jonasson and Willis 2011).

*Macronycteris commersoni* appeared to avoid longer torpor bouts when energy or water conservation were not vital. Instead, they entered the shortest torpor bouts of any heterotherm studied so far. All but three cave-dwelling individuals used micro-torpor bouts with patterns reflecting prevailing environmental conditions, appearing to be the preferred mode of torpor. Micro-torpor bouts were less frequent but longer during the dry season than the wet season. They were especially frequent in cave bats during the wet season and in the forest they were used in combination with an extended torpor bout on hot days (Reher and Dausmann 2021). Micro-torpor bouts combine an increased number of active phases, and thus higher vigilance, with energy and water savings. This could enable bats in exposed roosts in foliage to react quickly to threats and be beneficial in caves for maintaining social and territorial activities (Kunz and Lumsden 2003). However, micro-torpor usually occurred in a repetitive manner (12.0 ± 6.9 consecutive bouts), increasing not only the number of active periods but also the number of arousal phases. Arousing from torpor is potentially harmful, especially at low *T*_a_, because it increases oxidative stress and can cause cellular damage (Carey et al. 2003; Brown and Staples 2011; Nowack et al. 2019). Interestingly, Australian desert bats can arouse from torpor passively without active thermogenesis when *T*_a_ is near euthermic *T*_b_ (Bondarenco et al. 2013) and the energetic costs of rewarming from torpor for long-eared bats are reduced at higher *T*_a_ (Currie et al. 2014). Since *M. commersoni* MR during an arousal period rarely exceeded RMR, we assume that the costs of arousing at thermoneutrality are negligible, making micro-torpor an effective strategy.

Flexible torpor expression helped *M. commersoni* cope with different roosting conditions but torpor use in general comes with potential costs (reviewed in Landes et al. 2020). These include reduced responsiveness to the environment, missed opportunities for reproduction, reduced territory defence (Choi et al. 1998; Mzilikazi and Lovegrove 2002), cellular damage, diminished immune function and memory loss during hibernation (Millesi et al. 2001; Carey et al. 2003; Bouma et al. 2010). Energy and water can also be conserved by adjusting RMR; many mammals shift their TNZ in response to seasonal changes in *T*_a_ to reduce energy expenditure during euthermia (Lovegrove 2005). We did not find any differences between roosts or seasons in overall RMR or RMR at overlapping *T*_a_. This is unsurprising within the cave roost because it offers stable ambient conditions year-round. However, the lack of difference in RMR between the cave and forest roosts is unexpected, given the large fluctuations in *T*_a_ in the forest. The forest population has a TNZ between ~ 32–36 °C in the wet season (Reher and Dausmann 2021); while we were unable to determine the cave population's TNZ due to very stable conditions, we would suspect their TNZ to be close to the cave’s 32 °C. Thus, our measurements were probably below their thermal neutral conditions, suggested by the steeper slope in RMR. A more experimentally driven approach examining the bats’ responses under controlled conditions may consequently uncover geographical and seasonal variation in RMR.

Despite marked environmental differences between seasons and sites, bat body condition was similar among groups. Only forest-dwelling females had lower body condition than other bats, but sex was only a predictor for micro-torpor bout frequency and body condition only a predictor of extended torpor bout duration in the models. Since little is known about *M. commersoni* in general, we speculate that possibly younger, leaner females were present in the study or that reproduction is much more costly in the forest. We conducted the wet season measurements between mid-February and the end of March to keep seasonal life-history differences low, but it is likely that some females were still recuperating the costs of recent reproductive activities (in the cave, females wean their offspring in January; pers. obs.). In the wet season, DREE in the forest was ~ 1.6 times lower than in the cave and as low as in the cave in the dry season. This was largely due to the use of extended torpor as a response to heat; flexible torpor use dictated energy expenditure and not roosting conditions per se. For example, using several repeated micro-torpor bouts throughout the day reduced DREE by 46% compared to remaining euthermic; several micro-bouts combined with extended torpor bouts reduced DREE by 65% and entering a single, even longer extended torpor bout reduced DREE by 81%. Individual bats could balance DREE and body condition by adjusting bout duration and frequency. This strategy, combined with the insignificant costs of arousal from torpor at high *T*_b_, equips *M. commersoni* with a versatile physiological toolbox. Its broad repertoire allows the regulation of energy consumption and water depletion in direct response to prevailing conditions at a fine scale. This enables the species to effectively compensate for variable environmental pressures, and roost under contrasting ambient conditions, with little to no variation in body condition.

Our findings stress that physiological traits are not fixed within a species over seasonal and geographic scales. While there are many published studies of seasonal physiological variation (e.g. Brigham et al. 2000; Stawski and Geiser 2010; Czenze et al. 2017a), fewer examples exist investigating how separate populations of the same species cope with different environmental pressures (e.g., Dunbar and Brigham 2010; Stawski 2012; Noakes et al. 2016; Czenze et al. 2017b; van Jaarsveld et al. 2021). In a region like Madagascar, where almost 44% of the endemic vertebrate fauna are endangered or facing extinction (i.e., classified vulnerable or worse; IUCN 2021), it is of utmost importance to understand the entire range of variability within species’ ecophysiological traits. The variation in physiological traits that we observed for *M. commersoni*, for example, allows it to roost under vastly different environmental conditions, which has likely contributed to its successful colonisation of a range of habitat types distributed across almost all of Madagascar. Whether the variation uncovered in our study is related to phenotypic flexibility and can be expressed by individual *M. commersoni*, or whether these are local adaptations of each population, remains unclear (Geiser and Ferguson 2001; Dunbar and Brigham 2010). Nonetheless, our results demonstrate that conclusions drawn from limited datasets may not accurately represent a species as a whole. Studying more than one population and at different times of the year is logistically and financially challenging. However, this can illuminate intraspecific physiological, behavioural or morphological variation and ultimately give a clearer picture of a species’ potential for enduring a range of environmental pressures. Such insight is vital for predicting the consequences of disturbance events or rapid climatic changes and ensuring that conservation and species management actions do not fall short of their targets in regions with high environmental variability.

## Supplementary Information

Below is the link to the electronic supplementary material.

Supplementary file1 (PDF 770 KB)

## Data Availability

All data analysed during the current study are available from the corresponding author on reasonable request.
