# Supplementary material for: Disparate roost sites drive intraspecific physiological variation in a Malagasy bat
Source: Oecologia. 2021 Dec 24;198(1):35–52. doi: 10.1007/s00442-021-05088-2 (PMC8803705; doi:10.1007/s00442-021-05088-2)
Supplement: Supplementary file 1 — Supplementary file1 (PDF 770 KB) [file 442_2021_5088_MOESM1_ESM.pdf]

Supplementary material to manuscript:

Disparate roost sites drive intraspecific physiological variation in a Malagasy bat

Stephanie Reher, Hajatiana Rabarison, B. Karina Montero, James M. Turner and Kathrin H. Dausmann

Table S1. Detailed test-statistics of t-tests and Wilcoxon tests regarding the forearm length, body mass and body condition during torpid states of bats roosting in a cave during the dry and wet season as well as a forest in the wet season. Significance was adjusted through Bonferroni-Holm correction; bold values represent significant results.

**Variables tested**	**Test-statistics**	***p*-value**
Forearm length females		
Cave dry season vs. cave wet season	*W* = 21	0.529
Cave dry season vs. forest wet season	*W* = 0	**< 0.001**
Cave wet season vs. forest wet season	*W* = 0	**< 0.001**
Forearm length males		
Cave dry season vs. cave wet season	*W* = 11	0.914
Cave dry season vs. forest wet season	*W* = 0	**0.012**
Cave wet season vs. forest wet season	*W* = 0	**0.004**
Body mass females		
Cave dry season vs. cave wet season	*W* = 28	1
Cave dry season vs. forest wet season	*W* = 26.5	1
Cave wet season vs. forest wet season	*W* = 31	1
Body mass males		
Cave dry season vs. cave wet season	*W* = 15	0.61
Cave dry season vs. forest wet season	*W* = 2	0.059
Cave wet season vs. forest wet season	*W* = 10	0.061
Body condition females		
Cave dry season vs. cave wet season	*t* = 0.213	0.835
Cave dry season vs. forest wet season	*t* = 4.26	**0.009**
Cave wet season vs. forest wet season	*t* = 4.24	**0.005**
Body condition males		
Cave dry season vs. cave wet season	*t* = 0.273	1
Cave dry season vs. forest wet season	*t* = 0.200	1
Cave wet season vs. forest wet season	*t* = -0.160	1

Table S2. Rayleigh's test statistics, including sample sizes (N = individuals, n = pooled bouts across all individuals), describing the timing of entry into, and arousal from, extended and micro-torpor bouts in each season and roost site. ns indicates event timing that did not differ from a random distribution; bold values represent significant results.

	Time of day	Test-statistics (*r*)	*p*-value	Sample size
Extended torpor				
Cave, dry season, entry	ns	0.25	0.3234	N = 6, n = 19
Cave, dry season, arousal	ns	0.33	0.1537	
Cave, wet season, entry	09:21 ± 00:40 h	0.80	**0.006**	N = 6, n = 7
Cave, wet season, arousal	14:54 ± 00:29 h	0.89	**0.001**	
Forest, wet season, entry	10:23 ± 00:12 h	0.91	**< 0.001**	N = 14, n = 22
Forest, wet season, arousal	16:56 ± 00:17 h	0.96	**< 0.001**	
Micro-torpor				
Cave, dry season, entry	ns	0.05	0.2787	N = 9, n = 538
Cave, dry season, arousal	ns	0.06	0.1403	
Cave, wet season, entry	12:58 ± 00:05 h	0.34	**< 0.001**	N = 14, n = 475
Cave, wet season, arousal	13:18 ± 00:07 h	0.34	**< 0.001**	
Forest, wet season, entry	09:28 ± 00:09 h	0.20	**< 0.001**	N = 16, n = 456
Forest, wet season, arousal	09:43 ± 00:11 h	0.20	**< 0.001**	

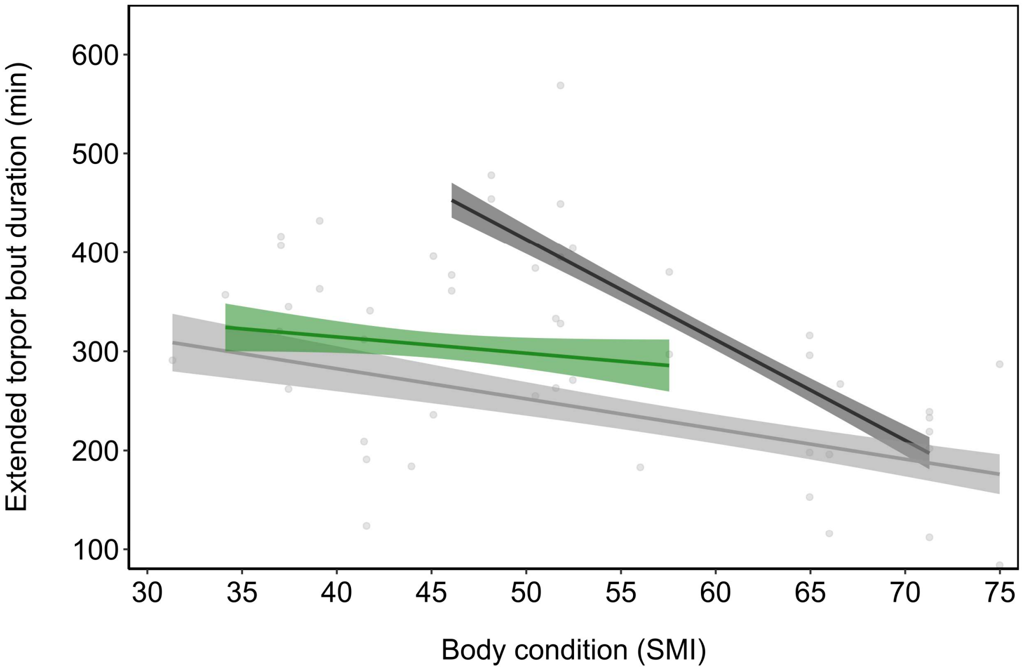

Figure S1. Estimated duration of extended torpor bouts (min) with 95% confidence interval (shaded area) as a function of body conditions (given as standardised mass index) of forest-dwelling bats (green) as well as cave-dwelling bats in the dry (dark grey) and wet season (light grey).
